# Rush Medical College

**Published:** 1872-02

**Authors:** 


### Rush Medical College.

We have received the annual announcement of the Rush Medical College, for the Spring Term of 1872. The term will begin Wednesday, March 6th, and will continue sixteen weeks to June 26th. Owing to the loss of the College building by the great fire the Faculty have secured the Lecture and Clinic Room of the Cook County Hospital, corner of Eighteenth and Arnold streets. Those desiring further information should address the Sec'y, C. T. Fenn, M. D., 1155 Michigan Avenue, Chicago.

